# *Salmonella* Enteritidis Isolate Harboring Multiple Efflux Pumps and Pathogenicity Factors, Shows Absence of O Antigen Polymerase Gene

**DOI:** 10.3389/fmicb.2016.01130

**Published:** 2016-08-03

**Authors:** Daniela Jones-Dias, Lurdes Clemente, Conceição Egas, Hugo Froufe, Daniel A. Sampaio, Luís Vieira, Maria Fookes, Nicholas R. Thomson, Vera Manageiro, Manuela Caniça

**Affiliations:** ^1^National Reference Laboratory of Antibiotic Resistances and Healthcare Associated Infections, Department of Infectious Diseases, National Health Institute Doutor Ricardo Jorge (INSA)Lisbon, Portugal; ^2^Centre for the Studies of Animal Science, Institute of Agrarian and Agri-Food Sciences and Technologies, University of PortoPorto, Portugal; ^3^Microbiology and Mycology Laboratory, Instituto Nacional de Investigação Agrária e VeterináriaLisbon, Portugal; ^4^Biocant, Parque Tecnológico de CantanhedeCantanhede, Portugal; ^5^Innovation and Technology Unit, Human Genetics Department, National Health Institute Doutor Ricardo Jorge (INSA)Lisbon, Portugal; ^6^Wellcome Trust Sanger Institute, Wellcome Trust Genome CampusHinxton, UK

**Keywords:** *Salmonella* Enteritidis, omphalitis, *wzy* deletion, epidemiology, pathogenicity factors, MGE, metal tolerance

## Background

*Salmonella enterica* is one of the most important causes of gastrointestinal infection in humans, being the great majority of infections related to the consumption of poultry meat and eggs (Foley and Lynne, 2008; EFSA/ECDC, 2015).

In animals, infections caused by serotype Enteritidis are rarely responsible for severe disease with animals frequently becoming asymptomatic carriers, except in the case of young chicks and poults, where outbreaks exhibiting clinical disease are often accompanied by high mortality rates (Foley et al., 2008, 2013). Indeed, *S. enterica* subsp. *enterica* serovar Enteritidis (*S*. Enteritidis) has been responsible for severe disease in industrial poultry farming facilities worldwide, posing a potential hazard for public health (Lutful Kabir, 2010).

In order to be infectious, *Salmonella* needs to adapt to different niches and conditions, where virulence and heavy-metal-tolerance factors play an important role, through co-selection events and the formation of pathogenicity islands, respectively (Hensel, 2004; Medardus et al., 2014). Furthermore, antibiotic resistance determinants can also facilitate their survival, with ubiquitous chromosomally encoded efflux mechanisms, playing an important role in both intrinsic, and acquired multidrug resistance. Other resistance mechanisms, such as changes in the membrane permeability, enzymatic modification, and target alterations may increase the levels of bacterial resistance, contributing to the success of the infection (Poole, 2004; Delmar et al., 2014; Li et al., 2015).

Both antibiotic susceptibility determination and serotyping constitute very useful tools for the epidemiologic classification of *S. enterica* isolates. Indeed, in *S. enterica*, the resistance rates fluctuate according to the serotype and with the antibiotic (Clemente et al., 2015). Classically, serotyping is based on the antigenic reactivity of lipolysaccharide (O antigen) and flagellar proteins (H antigen), followed by a designation using names or formulas (Grimont and Weill, 2007). In this study, we aimed to analyze the genome of a *S*. Enteritidis isolate responsible for omphalitis in chicks, exploring the molecular features associated with antibiotic resistance and pathogenicity, as well as the ability to spread the respective determinants.

## Methods

### Bacterial isolate, antibiotic susceptibility testing, and serotyping

The isolate (LV60) was recovered from a sample collected from the yolk sac of a chick with omphalitis, under the scope of the “*Salmonella* National Control Programme in food-producing animals and food of animal origin for bacteriological diagnosis, serotype identification and antibiotic susceptibility testing.” The guidelines of the Commission Decision (CD), 2007/407/EC were followed. LV60 was tested for its antimicrobial resistance through the determination of minimum inhibitory concentrations (MICs) using the agar dilution method, as previously described (Clemente et al., 2013) and according to the European Committee on Antimicrobial Susceptibility Testing (EUCAST) guidelines (http://www.eucast.org/). Briefly, a panel of 11 antibiotic compounds was tested in a 2-fold concentration series over the following ranges: ampicillin and tetracycline (0.5–64 μg/mL), gentamicin and trimethoprim (0.25–32 μg/mL), ciprofloxacin (0.008–8 μg/mL), cefotaxime (0.06–8 μg/mL), nalidixic acid and streptomycin (2–512 μg/mL), chloramphenicol (2–256 μg/mL), florfenicol (1–128 μg/mL) and sulphamethoxazole (8–1024 μg/mL). The epidemiological cut-off values recommended by EUCAST to *Salmonella* spp. were used for the interpretation of susceptibility testing results. Quality control was performed using the *Escherichia coli* ATCC 25922 strain. LV60 isolate was then serotyped by the slide agglutination method for its O and H antigens using the method of Kauffman-White scheme (Grimont and Weill, 2007).

### Whole genome sequencing (WGS), assembly, and annotation

Genomic DNA was extracted using DNeasy Blood and Tissue Kit (Qiagen), and DNA quantification was performed by Qubit Fluorometric Quantitation (Life Technologies), according to with the manufacturer's instructions. The genome was sequenced using a double strategy of 454 (Roche) and MiSeq (Illumina) sequencing.

Five hundred nanograms of bacterial DNA were fragmented by nebulization, followed by adaptor ligation to create double stranded DNA libraries and sequenced on a 454 GS FLX Titanium according to the standard manufacturer's instructions (Roche-454 Life Sciences). The second genome library was prepared from 1 ng of genomic DNA using the Nextera XT DNA Sample Preparation Kit (Illumina) and sequenced on the Illumina MiSeq sequencer (Illumina) using paired-end 2 × 150 bp reads.

First quality evaluation of raw read sequences and their corresponding quality values were assigned by the FastQC software. Reads were then trimmed and filtered according to quality criteria, and *de novo* assembled with Ray, version 2.3.1 (Boisvert et al., 2010). Contigs were searched for identity through blastn (http://blast.ncbi.nlm.nih.gov/Blast.cgi) against the nr/nt NCBI database to identify the closest bacterial genome and/or plasmid. Therefore, LV60 genome was mapped against the bacterial genome of *S*. Enteritidis strain p125109 and its plasmid (NC_011294 and HG970000, respectively) using GS Mapper version 2.9 (Roche). Additionally SNV (single nucleotide variants) and structural variants were also detected with the GS Mapper (Roche, version 2.9).

Structural and functional annotation was performed using PGP (Prokaryotic Genome Prediction) (Egas et al., 2014), an in-house developed pipeline. Taxonomy identification was performed by BLASTP search against the NCBI GenBank non-redundant (nr) database of the 16 s rRNA sequence gene, identified in the previous step and confirmed using RNAmmer v1.2 (Lagesen et al., 2007).

The final data was submitted in the DDBJ/EMBL/GenBank databases, using the Sequin software tool (http://www.ncbi.nlm.nih.gov/Sequin/). This dataset, which includes files in Genbank (LIHI01.1.gbff.gz), Fasta (LIHI01.1.fsa_nt.gz), and ASN.1 (LIHI01.1.bbs.gz) formats, can be accessed and/or reused at http://www.ncbi.nlm.nih.gov/nuccore/LIHI00000000.

### *In silico* analyses

CLC genomics workbench 8.0 (QIAGEN, Aarhus), PathogenFinder 1.1, ResFinder 2.1, PlasmidFinder 1.3, and MLST 1.8 (MultiLocus Sequence Typing) were used to estimate the number of pathogenicity determinants, acquired antibiotic resistance genes, plasmids and the MLST using the *S*. Enteritidis genome (Larsen et al., 2012; Zankari et al., 2012; Cosentino et al., 2013; Carattoli et al., 2014). SeqSero tool was used for *Salmonella* serotyping by whole genome sequencing (Zhang et al., 2015).

PHAST search web tool was applied to detect, identify and annotate prophage sequences (Zhou et al., 2011). ISsaga was used for the high throughput identification and semiautomatic annotation of insertion sequences in the genome (Varani et al., 2011). The presence of molecular determinants of antimicrobial resistance was predicted based on homology and SNP models using the Comprehensive Antibiotic Resistance Database (CARD; https://card.mcmaster.ca/analyze/rgi), through Resistance Gene Identifier software (RGI; McArthur et al., 2013).

## Results

LV60 isolate was serotyped as *S*. Enteritidis, using the method of Kauffman-White scheme, and found to be wild-type to all the antibiotics tested, except tetracycline.

The *de novo* assembly yielded 4.977 Mbp distributed in 83 contigs (largest contig with 970,921 bp) with a N50 of 491,005 bp. Overall, the structural and functional annotation with PGP detected 97 tRNA genes, 7 rRNA genes and identified 4656 mRNA genes.

From mapping against the bacterial genome of *S*. Enteritidis strain p125109, the main difference between the two genomes was the absence of the O-antigen polymerase gene *wzy* in the LV60 isolate, which in *S*. Enteritidis is located outside the O antigen gene cluster (Liu et al., 2014). The coding sequence of *wzy* gene was searched against the assembled genome using blastn, confirming its absence. The flanking regions of *wzy* gene, which coded for a disrupted membrane and a hypothetical protein, were also absent. The *wzy* gene is involved in the Wzx/Wzy-dependent pathway, which constitutes the predominant pathway for O-antigen production in Gram-negative bacteria, specifically in *Salmonella* (Hong et al., 2015).

However, in this study, the absence of the *wzy* gene did not compromised the use of a high-throughput genome sequencing serotype determination method (Zhang et al., 2015), which corroborated the result obtained by the gold standard method. Indeed, this method, based on the detection of O and H antigens encoding genes, predicted an antigenic profile 9:g,m:- based on the O-9,46 *wba*V gene, which encodes to the O-antigen tyvelosyl transferase. Furthermore, the *S*. Enteritidis serotype was confirmed by the presence of *sdf* gene (*Salmonella* difference fragment virulence gene), a characteristic marker of commonly circulating *S. enterica* serovar Enteritidis (Agron et al., 2001).

Sixty-one SNVs were detected between LV60 and the *S*. Enteritidis strain p125109. The SNVs that resulted in amino acid substitutions are represented in Table 1. *In silico* analysis with ResFinder tool did not reveal the presence of any acquired antibiotic resistance genes (90% identity and 40% minimum length) or plasmids (95% identity). However, the RGI analysis, using the *perfect algorithm*, showed the presence of a *Salmonella*-specific MerR-like gold (Au) sensor- GolS—involved in Au resistance (Pontel et al., 2007). This constitutes a matter of concern since antibacterial biocides and metals can contribute to the development and maintenance of antibiotic resistance in bacterial communities through mechanisms of cross- or co-resistance (Baker-Austin et al., 2006; Lemire et al., 2013; Pal et al., 2015).

**Table 1:** **Single nucleotide variants that represent amino acid substitutions in *S*. Enteritidis LV60 using *S*. Enteritidis strain p125109 as the reference genome**.

**Reference Position**	**Reference**	**Allele**	**Gene (Product)**	**Amino acid change**	**Coverage**
40158	C	T	SEN_RS00180 (arylsulfatase)	Pro92Ser	155
55278	C	A	*ileS* (isoleucine-tRNA ligase)	Ala557Glu	144
93979	G	A	SEN_RS00415 (hypothetical protein)	Ala96Thr	127
156264	G	A	SEN_RS00685 (peptidase M23)	Gly299Asp	123
353437	T	C	SEN_RS01600 (isopropylmalate isomerase)	Val454Ala	119
357149	A	T	SEN_RS01625 (hypothetical protein)	Leu1Met	177
401018	C	A	*prpE* (acetyl-CoA synthetase)	Arg9Ser	132
411602	T	G	SEN_RS01845 (hypothetical protein)	Trp209Gly	58
561577	T	C	SEN_RS02560 (MFS transporter)	Ser333Pro	68
659902	T	G	*dpiB* (sensor histidine kinase)	Tyr3Asp	52
988620	G	C	SEN_RS04610 (hypothetical protein)	Ala89Pro	130
1044895	G	T	*helD* (DNA helicase IV)/Mobile element	Ala204Ser	75
1156702	G	C	*sirA* (virulence gene transcriptional regulator)	Val181Leu	112
1325689	A	G	SEN_RS06450 (hydrogenase-1 operon protein HyaF)	Tyr209His	93
1427037	T	A	SEN_RS06930 (diguanylate phosphodiesterase)	Asp16Glu	92
1787654	A	G	SEN_RS08735 (transporter)	Arg348Gly	79
1807289	G	A	SEN_RS08820 (lipoprotein)	Ala14Val	79
1931818	C	T	SEN_RS09505 (NAD-dependent deacetylase)	Met37Ile	82
2115337	C	T	SEN_RS10585 (cobalamin biosynthesis protein CbiB)	Gly167Ser	104
2419980	G	A	SEN_RS11950 (NADH:ubiquinone oxidoreductase subunit M)	Leu474Phe	130
2426844	A	G	SEN_RS11980 (NADH dehydrogenase subunit G)	Val610Ala	125
2463887	T	C	SEN_RS12170 (amino acid transporter)	Ile452Val	34
2647060	G	A	SEN_RS12985 (outer membrane protein RatA)	Pro459Ser	108
2647626	G	T	SEN_RS12985 (outer membrane protein RatA)	Ala270Glu	111
2672592	A	C	SEN_RS13070 (hypothetical protein)	Ile313Ser	61
2956057	C	A	SEN_RS14420 (2-C-methyl-D-erythritol 4-phosphate cytidylyltransferase)	Arg53Leu	123
3185834	C	A	SEN_RS15495 (D-mannonate oxidoreductase)	Asn151Lys	81
3659470	G	T	SEN_RS17815 (membrane protein)	Gln71Lys	122
3802073	G	A	*coaD* (phosphopantetheine adenylyltransferase)	Val116Ile	127
4051393	T	C	SEN_RS19620 (DNase TatD)	Ser141Pro	150
4059155	G	A	*fadB* (3-ketoacyl-CoA thiolase)	Ala395Val	84
4348398	A	G	SEN_RS20980 (membrane protein)/ Salmonella Pathogenicity Island 4	Asn2902Asp	158
4402123	C	T	SEN_RS21190 (sugar:sodium symporter)	Ala350Val	77
4476625	T	C	SEN_RS21580 (hypothetical protein)	Lys76Glu	170
4555382	C	T	SEN_RS21985 (DNA polymerase III subunit chi)	Asp10Asn	110

Furthermore, the RGI strict algorithm, which detects previously unknown variants of known antimicrobial resistance genes, identified 52 genes involved in efflux, transport, and permeability, which might justify the low-level tetracycline resistance identified by phenotypic methods (Table 2). Resistance to additional classes of antibiotics such as fluoroquinolones, aminoglycosides, and chloramphenicol were bioinformatically predicted. Indeed, efflux pumps are often associated with discrete decreases in antibiotic susceptibility that may not necessarily reflect an alteration in interpretation categories (Fernández and Hancock, 2012). Genes responsible for the intrinsic resistance to benzylpenicillin, glycopeptides, macrolides, and rifampicin were also detected.

**Table 2:** **Perfect and strict best hit results, by predicted gene, obtained using the Resistance Gene Identifier (RGI)**.

**Predicted gene**	***e*-value**	**Identity (%)**	**Contig**	**Average coverage**	**Start**	**Stop**	**RGI Cut-off**	**RGI Protein Model_type**	**Antibiotic Resistance Ontology (ARO) category**
*golS*	1.41E–108	100	4	147.97	80575	81039	Perfect	homolog	efflux pump conferring AR; chloramphenicol RG; beta-lactam RG; gene modulating antibiotic efflux
*acrF*	0	99	4	147.97	73608	76775	Strict	homolog	efflux pump conferring AR; beta-lactam RG; fluoroquinolone RG
*sdiA*	0	99	2	127.7	1179091	1179813	Strict	homolog	chloramphenicol RG; gene modulating antibiotic efflux; fluoroquinolone RG; efflux pump conferring AR; tetracycline RG; rifampin RG; beta-lactam RG
*crp*	1.30E–151	99	7	160.37	388833	389465	Strict	homolog	efflux pump conferring AR; macrolide RG; beta-lactam RG; gene modulating antibiotic efflux; fluoroquinolone RG
*mdsA*	0	98	4	147.97	76772	77977	Strict	homolog	efflux pump conferring AR; chloramphenicol RG; beta-lactam RG
*mdsC*	0	98	4	147.97	72134	73624	Strict	homolog	efflux pump conferring AR; chloramphenicol RG; beta-lactam RG
*aac(6')-Iy*	2.36E–101	97	2	127.7	808040	808477	Strict	homolog	antibiotic inactivation enzyme; aminoglycoside RG
*cpxR*	1.24E–160	97	3	152.34	67603	68301	Strict	homolog	efflux pump conferring AR; aminocoumarin RG; aminoglycoside RG; gene modulating antibiotic efflux
*bacA*	0	97	14	155.64	142061	142882	Strict	homolog	peptide AR gene; gene conferring AR via molecular bypass
*cpxA*	0	96	3	152.34	66233	67606	Strict	homolog	efflux pump conferring AR; aminocoumarin RG; aminoglycoside RG; gene modulating antibiotic efflux
*baeR*	5.11E–165	96	2	127.7	107261	107983	Strict	homolog	efflux pump conferring AR; aminocoumarin RG; aminoglycoside RG; gene modulating antibiotic efflux
*emrY*	0	95	8	158.13	93935	95473	Strict	homolog	efflux pump conferring AR; tetracycline RG
*marA*	1.35E–82	95	2	127.7	702301	702690	Strict	homolog	chloramphenicol RG; gene modulating antibiotic efflux; gene modulating permeability to antibiotic; fluoroquinolone RG; efflux pump conferring AR; tetracycline RG; rifampin RG; beta-lactam RG
*H-NS*	9.89E–75	94	2	127.7	965098	965511	Strict	homolog	gene modulating antibiotic efflux; macrolide RG; fluoroquinolone RG; efflux pump conferring AR; tetracycline RG; beta-lactam RG
*mexD*	0	94	5	135.43	37513	40626	Strict	homolog	chloramphenicol RG; trimethoprim RG; macrolide RG; fluoroquinolone RG; efflux pump conferring AR; beta-lactam RG
*phoP*	6.18E–151	93	2	127.7	417112	417786	Strict	homolog	efflux pump conferring AR; polymyxin RG; macrolide RG; gene modulating antibiotic efflux; gene altering cell wall charge conferring AR
*emrR*	7.58E–115	93	8	158.13	92089	92619	Strict	homolog	efflux pump conferring AR; gene modulating antibiotic efflux; fluoroquinolone RG
*mexD*	0	93	4	147.97	209028	212177	Strict	homolog	chloramphenicol RG; trimethoprim RG; macrolide RG; fluoroquinolone RG; efflux pump conferring AR; beta-lactam RG
*mdtH*	0	92	2	127.7	349496	350704	Strict	homolog	efflux pump conferring AR
*mdtK*	0	92	2	127.7	607306	608679	Strict	homolog	efflux pump conferring AR; fluoroquinolone RG
*mexN*	0	92	2	127.7	113873	116995	Strict	homolog	efflux pump conferring AR; chloramphenicol RG
*mexN*	0	91	2	127.7	110792	113872	Strict	homolog	efflux pump conferring AR; chloramphenicol RG
*emrD*	0	90	7	160.37	11534	12718	Strict	homolog	efflux pump conferring AR
*mdtG*	0	90	2	127.7	339682	340896	Strict	homolog	efflux pump conferring AR
*pmrA*	1.77E–143	90	9	160.96	119082	119750	Strict	homolog	polymyxin RG; gene altering cell wall charge conferring AR
*emrA*	0	89	8	158.13	92719	93918	Strict	homolog	efflux pump conferring AR; fluoroquinolone RG
*pmrE*	0	89	2	127.7	174573	175739	Strict	homolog	polymyxin RG; gene altering cell wall charge conferring AR
*baeS*	0	89	2	127.7	107980	109383	Strict	homolog	efflux pump conferring AR; aminocoumarin RG; aminoglycoside RG; gene modulating antibiotic efflux
*tolC*	0	89	14	155.64	163404	164879	Strict	homolog	chloramphenicol RG; macrolide RG; fluoroquinolone RG; efflux pump conferring AR; aminocoumarin RG; tetracycline RG; rifampin RG; beta-lactam RG
*acrE*	0	88	1	155.02	4223	5380	Strict	homolog	efflux pump conferring AR; beta-lactam RG; fluoroquinolone RG
*mexD*	0	88	1	155.02	1098	4211	Strict	homolog	chloramphenicol RG; trimethoprim RG; macrolide RG; fluoroquinolone RG; efflux pump conferring AR; beta-lactam RG
*mdfA*	0	87	13	131.07	105101	106333	Strict	homolog	efflux pump conferring AR
*pmrF*	0	87	5	135.43	231615	232598	Strict	homolog	polymyxin RG; gene altering cell wall charge conferring AR
*mdtM*	0	86	11	163.1	148308	149549	Strict	homolog	efflux pump conferring AR
*ramA*	1.93E–71	86	4	147.97	311233	311622	Strict	homolog	chloramphenicol RG; gene modulating antibiotic efflux; gene modulating permeability to antibiotic; fluoroquinolone RG; efflux pump conferring AR; tetracycline RG; rifampin RG; beta-lactam RG
*mdtD*	0	86	2	127.7	109383	110795	Strict	homolog	efflux pump conferring AR
*acrA*	0	85	4	147.97	212200	213393	Strict	homolog	chloramphenicol RG; fluoroquinolone RG; efflux pump conferring AR; tetracycline RG; rifampin RG; beta-lactam RG
*phoQ*	0	85	2	127.7	415649	417112	Strict	homolog	efflux pump conferring AR; polymyxin RG; macrolide RG; gene modulating antibiotic efflux; gene altering cell wall charge conferring AR
*pmrB*	0	85	9	160.96	118002	119081	Strict	homolog	polymyxin RG; gene altering cell wall charge conferring AR
*mdtA*	0	82	2	127.7	116995	118332	Strict	homolog	efflux pump conferring AR; aminocoumarin RG
*pmrC*	0	82	9	160.96	119747	121390	Strict	homolog	polymyxin RG; gene altering cell wall charge conferring AR
*acrR*	1.83E–124	82	4	147.97	213535	214188	Strict	variant	chloramphenicol RG; gene modulating antibiotic efflux; fluoroquinolone RG; efflux pump conferring AR; antibiotic resistant gene variant or mutant; tetracycline RG; rifampin RG; beta-lactam RG
*robA*	0	81	11	163.1	77518	78387	Strict	homolog	chloramphenicol RG; gene modulating antibiotic efflux; fluoroquinolone RG; efflux pump conferring AR; tetracycline RG; rifampin RG; beta-lactam RG
*arnA*	0	79	5	135.43	229636	231618	Strict	homolog	polymyxin RG; gene altering cell wall charge conferring AR
*mdtL*	0	77	16	156.65	44691	45878	Strict	homolog	efflux pump conferring AR
*rosB*	0	74	4	147.97	230248	231924	Strict	homolog	polymyxin RG
*rosA*	0	71	4	147.97	232128	233348	Strict	homolog	efflux pump conferring AR; polymyxin RG
*rpoB*	0	58	19	154.2	4220	8248	Strict	variant	rifampin RG; antibiotic resistant gene variant or mutant
*katG*	0	56	3	152.34	121560	123740	Strict	variant	antibiotic resistant gene variant or mutant; isoniazid RG
*gyrB*	0	55	16	156.65	54369	56783	Strict	homolog	aminocoumarin RG; antibiotic resistant gene variant or mutant
*macB*	0	50	13	131.07	143618	145564	Strict	homolog	efflux pump conferring AR; macrolide RG
*vanG*	8.15E–81	38	4	147.97	113335	114447	Strict	homolog	glycopeptide RG; AR gene cluster, cassette, or operon; gene conferring AR via molecular bypass
*macA*	2.30E–51	35	13	131.07	142503	143621	Strict	homolog	efflux pump conferring AR; macrolide RG

*RG, resistance gene; AR, antibiotic resistance*.

The total number of pathogenicity determinants present in the genome of *S*. Enteritidis LV60, matching 1164 pathogenic families, showed a 94.1% certainty of the isolate being a human pathogen. Here we highlight the presence of *Salmonella* Pathogenicity Island 4, which usually encodes a non-fimbrial adhesion and the cognate type 1 secretion system (Gerlach et al., 2007).

The use of complementary web tools assigned this isolate to ST11, which according with MLST data (http://mlst.warwick.ac.uk/) is commonly found among CTX-M-14 and CTX-M-15-producing *S*. Enteritidis human isolates (Kim et al., 2011; Bado et al., 2012). In this study, the identification of ST11 in an isolate of animal origin, together with other pathogenicity determinants may suggest its zoonotic potential.

We also identified 6 prophage regions, among which three were incomplete and three were intact. The last included prophage regions reaching the lengths of 64.3, 49.2, and 31.7 Kb, and encoding 42, 78, and 66 DNA coding sequences, respectively.

Overall, 33 different IS were detected within the genome, which were distributed as follows: 27.03% of IS*3* family, 18.92% of IS*256* family, 13.51% of IS unclassified elements, 10.81% of IS*200*/IS*605* complex, and of IS*L3* family, 8.11% of IS*481* family, 5.41% of IS*630* family, and 2.7% of IS*1* and IS*110* families. All identified structures (pathogenicity island, prophages, ISs) constitute a multiplicity of pathogenicity factors in LV60 *S*. Enteritidis isolate and contribute for the fitness of the isolate in different environments; its presence may also suggest the possibility of acquisition of other factors by different mechanisms, including resistance genes e.g., by horizontal gene transfer, contributing to its biological diversity and genetic evolution.

## Conclusion

The detection of an avian *S*. Enteritidis isolate harboring multiple efflux pumps, pathogenicity factors, a variety of mobile genetic elements and heavy-metal-tolerance genes raises concerns regarding the dissemination of infection in birds and potential risk of zoonotic transmission.

This study demonstrated the added value of WGS as a routine tool for surveillance programs directed to food-producing animals, which might complement sanitary measures, essential to prevent the spread of *Salmonella* infections among animals. It also proved to have an added value as a complementary typing method. Moreover, the simultaneous detection of putative Au resistance, intrinsic antibiotic resistant genes, and mobile genetic elements, underline this method as a helpful resource to follow the spread and evolution of antibiotic resistance in this species by genomic comparison studies.

## Data access

This Whole Genome Shotgun project has been deposited at DDBJ/EMBL/GenBank under the accession LIHI00000000. The version described in this paper is version LIHI01000000.

## Author contributions

DJ designed the study, performed molecular experiments, analyzed the data and wrote the manuscript. LC performed the microbiological experiments and reviewed the manuscript. CE, HF performed 454 Roche genome sequencing experiments and analyze the data; DS, LV performed Illumina genome sequencing experiments. MF, NT analyzed the data. VM designed the study, analyzed the data and reviewed the manuscript. MC designed the study, reviewed and edited the manuscript. All authors read and approved the final manuscript.

## Funding

DJ has received research funding from Fundação para a Ciência e a Tecnologia (FCT, grant number SFRH/BD/80001/2011). VM was supported by FCT fellowship (grant SFRH/BPD/77486/2011), financed by the European Social Funds (COMPETE-FEDER) and national funds of the Portuguese Ministry of Education and Science (POPH-QREN). We thank the support of FCT grant number PEst-OE/AGR/UI0211/2011-2014 and UID/MULTI/00211/2013.

### Conflict of interest statement

The authors declare that the research was conducted in the absence of any commercial or financial relationships that could be construed as a potential conflict of interest.
